# Disease-specific survival of malignant melanoma after Mohs micrographic surgery is not impacted by initial margins: A systematic review and meta-analysis

**DOI:** 10.1016/j.jdin.2023.06.009

**Published:** 2023-06-28

**Authors:** Olivia M. Crum, Elliott H. Campbell, Cynthia J. Chelf, Addison M. Demer, Jerry D. Brewer

**Affiliations:** a Department of Dermatology, Mayo Clinic School of Graduate Medical Education, Rochester, Minnesota; b Mayo Clinic Libraries, Rochester, Minnesota; c Division of Dermatologic Surgery, Department of Dermatology, Mayo Clinic, Rochester, Minnesota

**Keywords:** disease-specific mortality, initial margin, melanoma, Mohs micrographic surgery, survival

## Abstract

**Background:**

During Mohs surgery for melanoma, evidence has demonstrated that many surgeons opt for smaller initial margins than traditionally recommended (0.5 cm for in situ and 1 cm for invasive). Literature regarding surgical outcomes based on initial margin is sparse.

**Objective:**

To determine differences in disease-specific survival of melanoma after Mohs micrographic surgery for varied initial surgical margins.

**Methods:**

A literature search was conducted on February 14, 2022, from MEDLINE via PubMed (1946-present), Embase (1974-present), Central (1991-present), and Scopus (1960-present). The primary outcome was disease-specific mortality.

**Results:**

Nineteen studies were included for final analysis. The overall disease-specific mortality rate of melanoma in all included studies was 0.5% (CI, 0.1-0.8; *P*, .010). Disease-specific mortality for 1 to 5, 5, and 6 to 10 mm categories were 0.4% (CI, 0.0-0.9; *P*, .074), 0.7% (CI, 0.2-1.3; *P*, .2-1.3), and 0.4% (CI, –0.9 to 1.8; *P*, .524), respectively. None of the variances across initial margin categories were statistically significant.

**Limitations:**

Early-stage melanomas have low overall mortality rates. In our associated article, initial margins of 5 to 10 mm were shown to have the lowest rates of local recurrence.

**Conclusions:**

In this systematic review and meta-analysis, melanoma-specific mortality was not significantly impacted by the initial surgical margin taken during Mohs micrographic surgery.

**Capsule Summary**

• Literature regarding surgical outcomes based on initial margin during Mohs micrographic surgery is sparse.
• In this meta-analysis, melanoma-specific mortality was not significantly impacted by the initial surgical margin taken during Mohs micrographic surgery. In our associated article, local recurrence was shown to be lowest with initial margins of 5 to 10 mm.

## Introduction

The use of Mohs micrographic surgery (MMS) in the treatment of melanoma has steadily increased.^1^ The National Comprehensive Cancer Network guidelines now support the selective consideration of MMS, or other surgical methods that provide comprehensive histologic assessment, for minimally invasive (T1a) melanomas in anatomically constrained areas (Version 3.2022 National Comprehensive Cancer Network guidelines on cutaneous melanoma).^2^ Recent studies have shown equivalent-to-moderately-improved survival and lower local recurrence rates for melanoma treated with MMS compared to wide local excision (WLE).^3^, ^4^, ^5^, ^6^

Surgical techniques for MMS of melanoma vary widely across the United States, without expert consensus guidelines.^7^^,^^8^ This includes selection of the initial surgical margin, which is influenced by anatomic location, proximity to free margin, cosmetic distortion, and other potential constraints. The “standard margin” for melanoma in situ (MMIS) and invasive melanoma (Breslow depth ≤1.0 mm) treated with WLE is generally considered to be 5-mm and 10-mm, respectively (*American* *Academy of* *Dermatology* *12: Melanoma: Appropriate Surgical Margins—National Quality Strategy Domain: Patient Safety*).^9^ There is a paucity of data on appropriate surgical margins for special site melanomas treated with MMS; evidence has shown that Mohs surgeons frequently deviate from standard WLE margins.^7^^,^^8^^,^^10^

To our knowledge, there is no previous literature comparing surgical outcomes of melanoma after MMS based on initial margin size, aside from a similar paper published by our group which analysis local recurrence after MMS based on initial margin.^11^ In this study, a systematic review and meta-analysis was conducted to compare the disease-specific survival rates of malignant melanoma after MMS based on initial margin.

## Methods

This study was performed in accordance with a protocol that prespecified the study criteria, including study selection, inclusion/exclusion criteria, data extraction, statistical analysis, heterogeneity, and measurements of inconsistencies. The methods employed in this manuscript are in accordance with the Cochrane collaboration guidelines (www.cochrane.org), quality of reporting of meta-analyses statement, and the Newcastle Ottawa method of quality assessment.^12^

### Literature search

With the assistance of a professional librarian, a literature search was conducted on February 14, 2022, from multiple databases including MEDLINE via PubMed (1946-present), Embase (1974-present), Central (1991-present), and Scopus (1960-present). Subject headings and keywords were used for the concepts of MMS, surgical margin, recurrent disease, and melanoma. All studies that met these search criteria were included.

### Inclusion and exclusion criteria

Study inclusion criteria required surgical management with en face histologic assessment of melanoma with a set initial margin (including ranges of initial margins). Disease-specific mortality data was required. Single subject case reports were excluded. Abstracts were included. Non-English articles were excluded due to inability to properly translate and find usable data. No contact with authors was required. National database studies and studies with redundant data were excluded. In cases of redundant data, the study with the highest number of usable subjects was selected for inclusion.

### Study selection

Studies were independently reviewed by 2 coinvestigators (E.H.C. and O.M.C.) with any discrepancies resolved by senior authors (J.D.B. and A.M.D.).

### Study quality assessment and risk of bias

Quality assessment was performed on all included articles. The Newcastle-Ottawa quality assessment tool was utilized for observational cohort studies to determine overall risk of bias.^13^ Table I outlines the quality assessment for cohort studies and associated margins. A separate methodological quality and synthesis tool was used for single arm studies.^21^ Table II outlines the quality assessment for single-arm studies and associated margins.

**Table I:** Quality assessment of the cohort studies

Author (y)	Representativeness of exposed cohort	Selection of nonexposed	Ascertainment of exposure	Outcome of interest study start	Comparability	Assessment of outcomes	Length of follow-up	Adequacy of follow-up
Chin-Lenn (2013)^14^	∗		∗	∗	N/A	∗	∗	∗
Demer (2019)^15^	∗	∗	∗	∗	N/A	∗	∗	∗
Jahn (Feb 2006)^16^			∗	∗	N/A	∗	∗	∗
Jahn (Jul 2006)^17^		∗	∗	∗	N/A	∗	∗	∗
Moehrle (2006)^18^	∗	∗	∗	∗	N/A	∗	∗	∗
Nosrati (2017)^19^	∗	∗	∗	∗	N/A	∗	∗	∗
Walling (2007)^20^	∗	∗	∗	∗	N/A	∗	∗	∗

Presence of “∗” indicates the study has adequately met criteria for the indicated quality assessment measure.

**Table II:** Quality assessment of the single arm studies

Author (y)	Selection	Exposure adequately ascertained	Outcome adequately ascertained	Alternate causes	Challenge/rechallenge phenomenon	Dose-response effect	Length of follow-up	Reproducibility
Bhardwaj (2006)^22^	∗	∗	∗	N/A	N/A	N/A	∗	∗
Bienert (2003)^23^	∗	∗	∗	N/A	N/A	N/A	∗	∗
Degesys (2019)^24^	∗	∗	∗	N/A	N/A	N/A	∗	∗
deVries (2016)^25^	∗	∗	∗	N/A	N/A	N/A	∗	∗
Foxton (2019)^26^	∗	∗	∗	N/A	N/A	N/A		∗
Kunishige (2019)^10^	∗	∗	∗	N/A	N/A	N/A	∗	∗
Lawrence (2014)^27^	∗	∗	∗	N/A	N/A	N/A	∗	∗
Lee (2008)^28^	∗	∗	∗	N/A	N/A	N/A	∗	∗
Moller (2009)^29^	∗	∗	∗	N/A	N/A	N/A		∗
Shumaker (2009)^30^		∗	∗	N/A	N/A	N/A	∗	∗
Temple (2006)^31^	∗	∗	∗	N/A	N/A	N/A	∗	∗
Then (2009)^32^		∗	∗	N/A	N/A	N/A	∗	∗

Presence of “∗” indicates the study has adequately met criteria for the indicated quality assessment measure.

### Data abstraction and management

The primary outcome for this systematic review and meta-analysis was disease-specific mortality rates of melanoma (invasive and in situ) after surgical management with en face histologic assessment. Mortality data were separated out from the concomitantly extracted recurrence data (presented in a separate manuscript). The reasoning behind this is that after data collection but before analysis, it was determined that the recurrence and mortality data required separate groupings of margin categories to be inclusive of articles and granular in nature. “Slow Mohs” and staged excisions were included if en face processing was utilized. The initial surgical margin included any clinically tumor-free margin, including any margin taken in the debulking layer. Disease-specific mortality was determined using a Freeman-Tukey transformed proportion analysis (random effects model). All data were abstracted and analyzed using OpenMeta statistical software.^33^ The Meta-analysis of Observational Studies in Epidemiology reporting guidelines were followed.

### Selection of margin categories

Margin categories were created based on the need to include all studies, with some studies having ranges of initial margins. The initial margin categories are 1 to 5 mm (excluding studies with exactly 5 mm margin), 5 mm, and 6 to 10 mm.

### Assessment of heterogeneity

Heterogeneity was assessed via the Iˆ2 statistic and reported in the figures below. Heterogeneity is deemed low if Iˆ2 was less than 25%, medium if between 25% to 75%, and high if greater than 75%.

## Results

### Description of included studies

**Fig 1:**
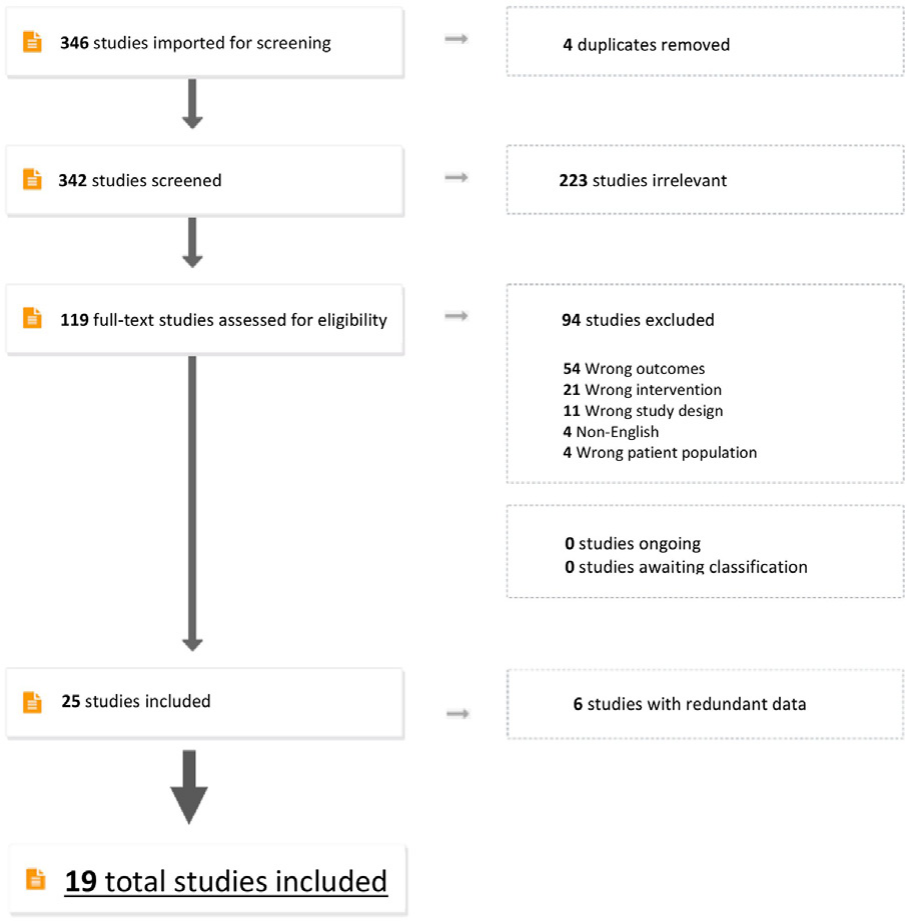
Preferred Reporting Items for Systematic Reviews and Meta-Analyses (PRISMA) diagram illustrating studies included.

A total of 342 studies were included for review, after removal of duplicates. After abstract review, 119 full-text articles were assessed via full-text review for eligibility. A total of 94 studies were excluded, in decreasing order, due to wrong outcomes (54), wrong intervention (21), wrong study design (11), non-English (4), and wrong patient population (4). Among the 119 full-text review articles, 25 studies met inclusion criteria. After elimination of studies with redundant data, a total of 19 studies were included for final analysis. A Preferred Reporting Items for Systematic Reviews and Meta-Analyses (PRISMA) flow-chart is found in Fig 1. Cohen’s Kappa was 0.83.

### Disease-specific mortality

Table III lists the disease-specific mortality rates of each category with associated CI and *P* values.

**Table III:** Results of meta-analysis

Invasive/in situ status	Margin category (mm)	Disease-specific mortality (%)	Confidence interval	*P* value	Heterogeneity *P* value
Both	Overall	0.5	0.1-0.8	.010	.140
	1-5	0.4	0.0-0.9	.074	.929
	5	0.7	0.2-1.3	.012	.360
	6-10	0.4	−0.9 to 1.8	.524	.166
In situ	Overall	0.0	0.0-0.1	.304	.809
	1-5	0.5	−0.1 to 1.0	.089	.971
	5	0.8	−0.1 to 1.6	.077	.937
	6-8	0.0	0.0-0.1	.460	.618
Invasive	Overall	0.9	−0.1 to 1.9	.073	.297
	1-5	1.5	−0.4 to 3.5	.127	.940
	5	0.8	−0.6 to 2.2	.273	.124
	6-10	13.6	−7.0 to 34.2	.195	.160

#### Overall mortality

**Fig 2:**
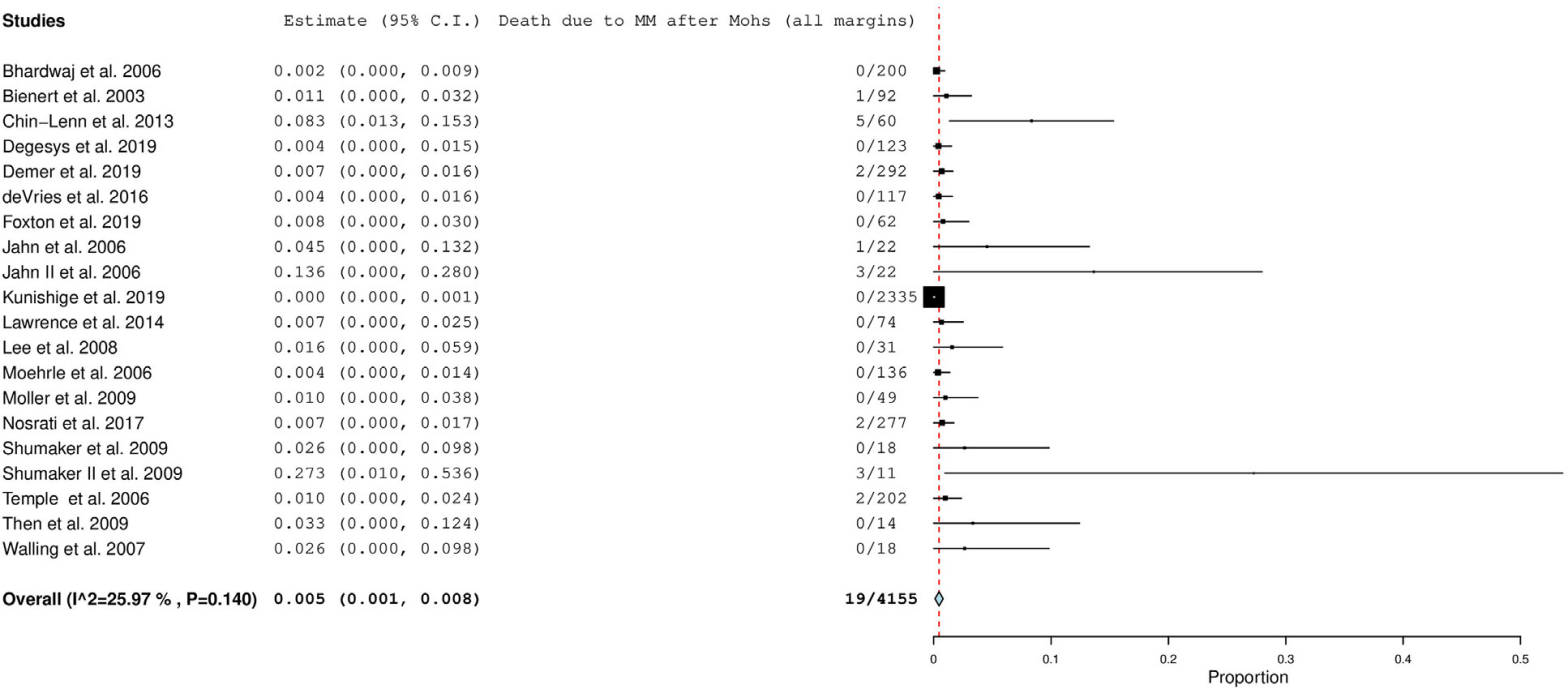
Overall disease-specific mortality rate. The *red dotted line* is an extension of the average of the overall pooled effect from the included studies. Each *black line* in the graphical display represents a study. The midpoint of the black box symbolizes the point estimate of the effect and its size is proportionate to the weight of the study. Studies that have a larger N provide more information and are therefore allotted greater weight. The diamond below the studies represents the overall pooled effect from the included studies. The width of the diamond shows the confidence interval for the overall effect.

Overall disease-specific mortality for all studies was 0.5% (CI, 0.1-0.8; *P*, .010). Mean follow up of all studies was 59 months. Disease-specific mortality did not vary significantly between initial margin categories. Fig 2 displays the overall forest plot.

(Adapted from “Seeing the Forest by Looking at the Trees: How to Interpret a Meta-Analysis Forest Plot” PMID: 33939533)

**Fig 3:**
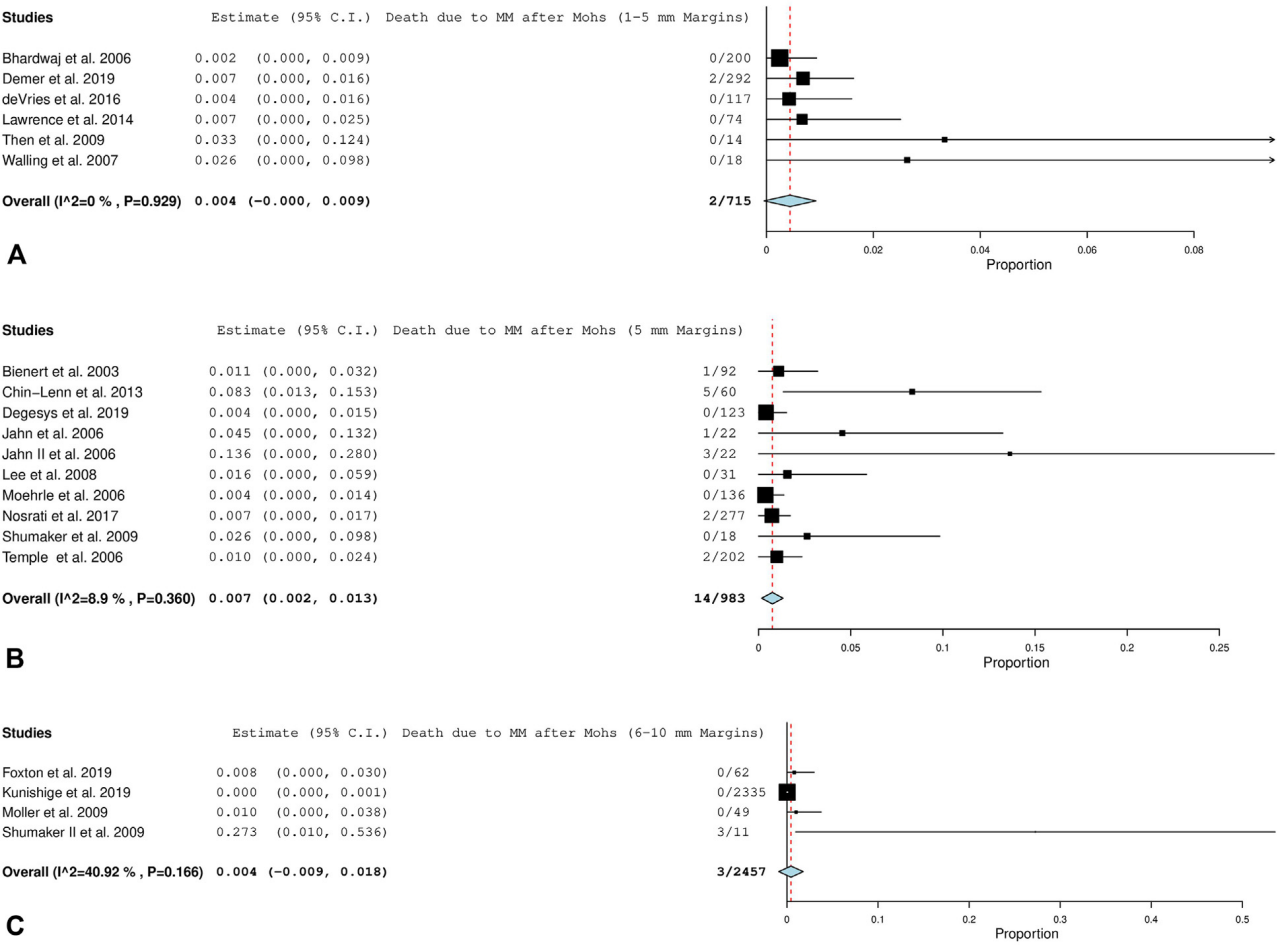
**A,** Disease-specific mortality after 1 to 5 mm margins. **B,** Disease-specific mortality after 5 mm margins. **C,** Disease-specific mortality after 6 to 10 mm margins. The *red dotted line* is an extension of the average of the overall pooled effect from the included studies. Each *black line* in the graphical display represents a study. The midpoint of the black box symbolizes the point estimate of the effect and its size is proportionate to the weight of the study. Studies that have a larger N provide more information and are therefore allotted greater weight. The diamond below the studies represents the overall pooled effect from the included studies. The width of the diamond shows the confidence interval for the overall effect.

Fig 3, *A*-*C* display the forest plots for each margin category subgroup.

(Adapted from “Seeing the Forest by Looking at the Trees: How to Interpret a Meta-Analysis Forest Plot” PMID: 33939533)

#### MMIS mortality

**Fig 4:**
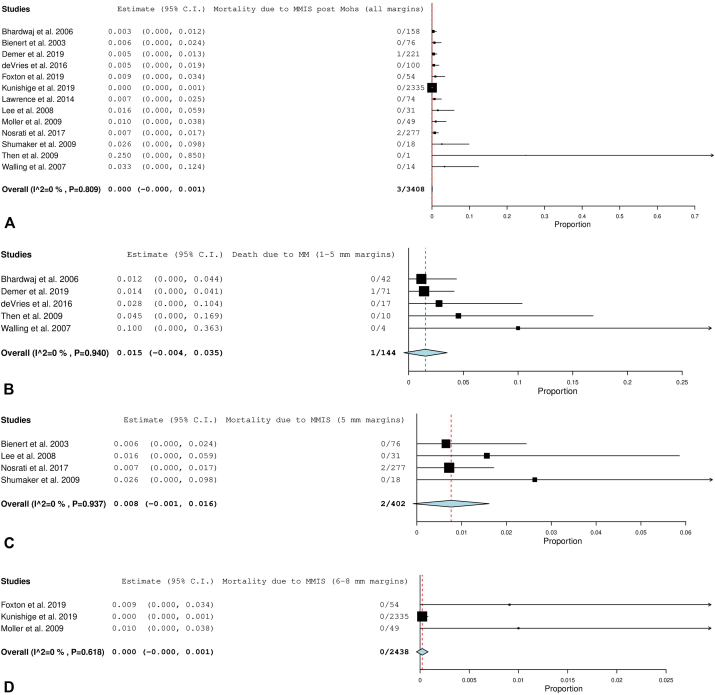
**A,** Disease-specific mortality after all margins for melanoma in situ (MMIS). **B,** Disease-specific mortality after 1-5 mm margins for MMIS. **C,** Disease-specific mortality after 5 mm margins for MMIS. **D,** Disease-specific mortality after 6 to 8 mm margins for MMIS. The *red dotted line* is an extension of the average of the overall pooled effect from the included studies. Each *black line* in the graphical display represents a study. The midpoint of the black box symbolizes the point estimate of the effect and its size is proportionate to the weight of the study. Studies that have a larger N provide more information and are therefore allotted greater weight. The diamond below the studies represents the overall pooled effect from the included studies. The width of the diamond shows the confidence interval for the overall effect. *MMIS*, Melanoma in situ.

Overall disease-specific mortality for all margins in the MMIS subgroup analysis was 0.0% (CI, 0.0-0.1; *P*, value .304). Fig 4, *A*-*D* display the forest plots for each of these groups.

(Adapted from “Seeing the Forest by Looking at the Trees: How to Interpret a Meta-Analysis Forest Plot” PMID: 33939533)

#### Invasive mortality

**Fig 5:**
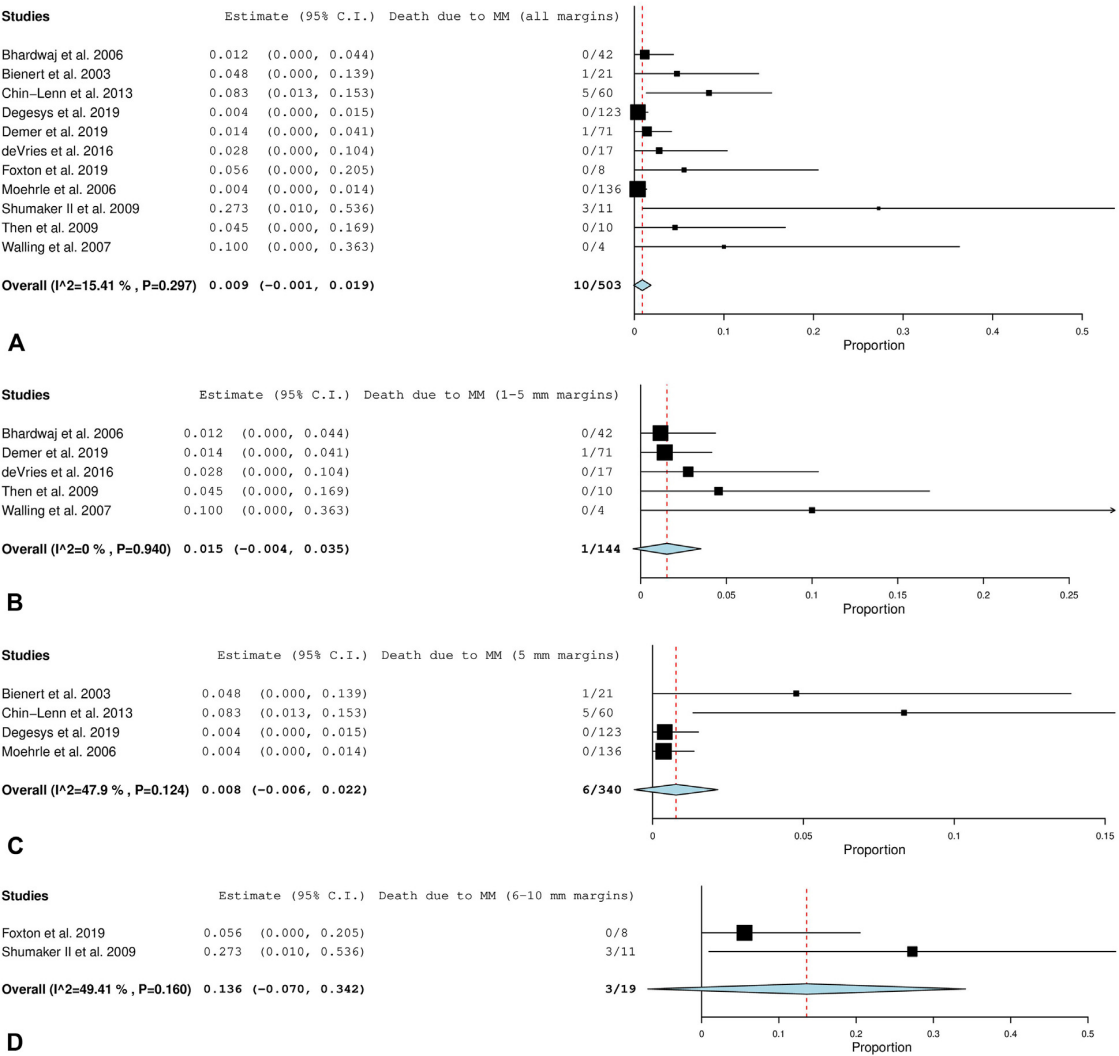
**A,** Disease-specific mortality after all margins for invasive melanoma. **B,** Disease-specific mortality after 1 to 5 mm margins for invasive melanoma. **C,** Disease-specific mortality after 5 mm margins for invasive melanoma. **D,** Disease-specific mortality after 6 to 10 mm margins for invasive melanoma. The *red dotted line* is an extension of the average of the overall pooled effect from the included studies. Each *black line* in the graphical display represents a study. The midpoint of the black box symbolizes the point estimate of the effect and its size is proportionate to the weight of the study. Studies that have a larger N provide more information and are therefore allotted greater weight. The diamond below the studies represents the overall pooled effect from the included studies. The width of the diamond shows the confidence interval for the overall effect.

Overall disease-specific mortality for all margins in the invasive subgroup analysis was 0.9% (CI, –0.1 to 1.9, *P*, .073). The 6 to 10 mm margin category subset demonstrated a considerably higher mortality rate at 13.6%. This was a small group of patients (*n* = 19), and most were periocular cases. Fig 5, *A*-*D* display the forest plots for each of these groups.

(Adapted from “Seeing the Forest by Looking at the Trees: How to Interpret a Meta-Analysis Forest Plot” PMID: 33939533)

### Follow-up

The overall follow-up for all studies was 59.90 months. The mean follow-up time for the 1 to 5, 5, and 6 to 10 mm categories was 36.99, 58.31, and 67.39 months, respectively.

## Discussion

To our knowledge, this is the first study to compare disease-specific mortality rates of melanoma after MMS based on initial margin size. This study demonstrated that the melanoma-specific mortality after MMS was not significantly impacted by initial surgical margins.

Our findings are consistent with previous literature, including an analysis of the Surveillance, Epidemiology, and End Results database that demonstrated a 5-year melanoma-specific mortality rate of 0.2% for melanoma after MMS.^34^ The overall melanoma-specific mortality rate in the current study was 0.5%.

In most studies of Mohs for invasive melanoma, the operating Mohs surgeon began with a margin less than that traditionally recommended for WLE (with permanent histologic evaluation and bread loaf sections). The lack of survival difference between initial margin groups (1-5, 5, 6-10) suggests that Mohs may be safely performed with narrower-than-recommended initial surgical margins without compromising survival outcomes.

When selecting an initial surgical margin, there are numerous factors to take into consideration. In a previous study conducted by our team, the local recurrence rates of melanoma were analyzed based on the initial margin size,^11^ discovering that the lowest local recurrence rates were associated with initial margins ranging from 5 to 10 mm. Consequently, it was recommended to consider an initial margin of 5 to 10 mm, provided that other factors such as tumor characteristics, anatomical or functional considerations permit. Our current mortality data shows no significant difference across initial margin categories, further supporting the conclusion that a 5- to 10-mm initial margin should be considered when other factors allow. However, future prospective studies or randomized control trials comparing initial margins are needed.

Limitations of our study included the predominant retrospective nature of the studies included in our systematic review and meta-analysis. There were no studies that compared different initial margins within the same study. There was a paucity of studies with 10 mm initial margin (generally considered standard margin for invasive tumors with Breslow depth of ≤1.0 mm) and a complete lack of studies >10 mm. Due to the inclusion of several studies with ranges of initial margins, the current study design was unable provide more granular margin categories without excluding a large number of subjects.

Despite conducting a large systematic review, the difference in mortality values in our study were nonsignificant, when compared across different initial margins taken. Melanoma-specific mortality is still a rare occurrence for MMIS and early invasive melanoma.

Follow-up was a mean of 59 months. For all aggregated cases of MMIS (3408 tumors) in our meta-analysis, there were only 3 deaths attributed to melanoma. This resulted in a mortality rate of 0.0% (CI, 0.0-0.1; *P*, value .304). For the invasive subgroup analysis, the study did not consider the stage/Breslow thickness. There were multiple studies without any instances of mortality. As such, future studies, with longer follow-up times and a larger collection of tumor data, will be necessary to further elucidate any trends of significance.

The original search criteria focused on recurrence rates as they pertain to the initial surgical margin. As the data was abstracted and analyzed, it became apparent that the mortality data associated with initial surgical margin status required separate margin categories to be as inclusive as possible with articles that met inclusion criteria. Thus, the decision was made to publish as 2 separate articles. However, given the initial search strategy focused on recurrence rates, it is possible that there are some articles that focus on mortality rates associated with initial margins employed during MMS that could have been missed. We feel this to be very unlikely given the limited number of papers published on the topic of MMS in the setting of melanoma, especially those that include and present the initial margin taken. The authors feel confident this dataset represents a true comprehensive list of inclusion articles that have been published on the topic.

Studies had variable utilization of immunohistochemistry staining and often did not report if immunohistochemistry was used. There was heterogeneity in techniques, including modified and slow-Mohs, and tumor types. It is likely that some studies did not use the exact listed initial margin in all instances. Due to the nature of MMS, the initial clinical margin in each study may not correspond to the final histologic margin or volume of tumor extirpated.

## Conclusion

In this systematic review and meta-analysis, melanoma-specific mortality was not significantly impacted by the initial surgical margin taken during MMS. However, in our related article, initial margins of 5 to 10 mm were shown to have the lowest rates of local recurrence. These collective findings should be considered when determining the appropriate initial surgical margin for Mohs treatment of melanoma.

## Conflicts of interest

None disclosed.
